# Evaluating pediatric peripheral neuromuscular disorders using deep neural networks on electrodiagnostic data

**DOI:** 10.1016/j.cnp.2025.08.001

**Published:** 2025-10-03

**Authors:** G.K. Cooray, L. Nastasi, D. Motan, J. Deeb

**Affiliations:** a Great Ormond Street Hospital, Great Ormond Street, London, WC1N 3JH, UK; b Karolinska Institutet, Stockholm, 171 77, Sweden; c UCL Great Ormond Street Institute of Child Health, London, WC1N 1EH, UK; d Queens Hospital, Romford, Greater London, RM7 0AG, UK

**Keywords:** EMG, Neural networks, AI, Diagnostic accuracy

## Abstract

**Objective:**

This study aims to assess application of deep neural networks to predict neuromuscular disorders in patients based on electrodiagnostic data and compare with clinical assessment.

**Methods:**

Patients evaluated with electrodiagnostic tests in intensive care over a 10-year period were included in this study. The data set contained both electrodiagnostic and clinical information. Based on the final diagnosis, patients were classified into six groups: non-primary neuromuscular disorders, neuropathy, motor-neuronopathy, myopathy, neuromuscular-junction disorders, and critical-illness neuromyopathy. The neural network was trained on the data.

**Results:**

The data set was small, allowing training of the neural network on a standard laptop. The validation results were promising, with an accuracy of 0.92, an ROC-AUC of 0.99, and a precision recall AUC of 0.97. The confusion and positive predictive value matrix demonstrated high performance, with diagonal values exceeding 0.82.

**Conclusion:**

This study demonstrates the efficacy of neural networks in predicting neuromuscular disorders using electrodiagnostic tests. The performance of the model was comparable to clinical assessment. These findings suggest that with more extensive datasets, neural networks can provide reliable estimates of neuromuscular diagnoses.

**Significance:**

Incorporating neural networks into diagnostic workflows could enhance decision-making, especially in scenarios requiring reassessment or complementary investigations.

## Highlights

• Predicting diagnosis using Deep Neural Networks classification of electrodiagnostic and clinical data.
• Deep Neural Networks show diagnostic performance similar to experienced physicians.
• Further data sets could be used to create models as diagnostic adjuncts for physicians.

## 1 Introduction

This research explores the application of artificial intelligence (AI) techniques for analyzing electrodiagnostic (EDX) and clinical data to forecast patient diagnoses. Traditionally, this responsibility falls to medical professionals specializing in neurology and neurophysiology due to the intricate nature of the work, which demands significant training and extensive practical experience (Guérit et al., 2009, Pitt, 2011, Pitt, 2012). The task becomes particularly demanding in intensive care units (ICUs), where patients may present with a variety of conditions stemming from trauma, genetic disorders, acquired disorders, or infections (David and Jones Jr., 1994, Kasinathan et al., 2021). Genetic factors, in particular, are more prevalent in pediatric cases, often leading to complex clinical presentations (Menezes and North, 2012).

Evaluating such cases necessitates a multifaceted approach that includes clinical exams, laboratory tests, imaging, and electrodiagnostic studies like nerve conduction studies and electromyography (EMG). This study used EDX and clinical data from all patients undergoing electrodiagnostic examinations in the ICUs of a tertiary pediatric center over a decade, presented previously in Nastasi et al. (2025). In summary, it was shown that combining EDX and clinical data it is possible to predict final diagnoses at a satisfactory level—a conclusion often assumed in clinical practice and corroborated by other studies, both in adult and pediatric population (Nastasi et al., 2025, Pitt, 2011, Preston and Shapero, 2005)

In healthcare, AI is advancing rapidly, including in diagnostic applications across both structural and physiological domains (Chan et al., 2020, Roy et al., 2019). Numerous studies have focused on converting raw data features into meaningful parameters for detecting abnormalities within diagnostic datasets (Goodfellow, 2016, de Jonge et al., 2024, Craik et al., 2019). These efforts often require complex methods to interpret features derived from imaging datasets or time-series data (Goodfellow, 2016). For example, intrinsic data structures are often analyzed using techniques ranging from basic visualizations, such as histograms and bar charts, to more sophisticated approaches like principal component analysis (PCA) or t-distributed stochastic neighbor embedding (Hotelling, 1933, van der Maaten and Hinton, 2008).

The t-distributed Stochastic Neighbor Embedding (t-SNE) algorithm is a dimensionality reduction technique that enables complex, high-dimensional data to be visualized in two or three dimensions. It works by preserving the relative similarities between data points, using probability distributions to reflect how close points are to each other in both the original and reduced-dimensional spaces. To address the common “crowding” issue in low-dimensional projections, t-SNE employs a Student’s t-distribution, which better separates clusters in the visualization. While t-SNE is useful for identifying patterns and groupings within the data, it is not a classification tool. For that purpose, predictive models such as regression algorithms or machine learning frameworks are needed to assign diagnostic categories to new data.

A wealth of literature supports the use of deep neural networks (DNN) for classifying time-series data, often employing supervised or semi-supervised methodologies. Neural network structures, such as convolutional neural networks (CNNs), recurrent neural networks (RNNs), and generative adversarial networks (GANs), are frequently applied to time-series data like brain signals (EEG) (Roy et al., 2019, Bai et al., 2018, Bashivan et al., 2015, Habashi et al., 2023). CNNs, in particular, are widely used for image-based diagnostic tasks (Ghafoorian et al., 2017). Recent studies indicate that DNNs can identify statistical correlations within datasets independently, eliminating the need for preprocessing that other techniques require. This streamlines the data preparation process for classification. Consequently, the resulting pipelines can accommodate a broader range of input data types.

In this study, we analyzed processed EMG data, evaluating each patient according to standard neurophysiology definitions, and applied EDX diagnostic criteria in conjunction with clinical data to establish the final diagnosis. Similar studies have been conducted by other researchers, who often employ neural networks and decision tree methods (Maleki Varnosfaderani and Forouzanfar, 2024, Goodfellow, 2016). This research specifically employed DNNs due to their effectiveness in complex pattern recognition tasks.

This study investigated a large pediatric ICU dataset, utilizing a DNN to evaluate diagnostic predictive accuracy in comparison to standard clinical assessment based on EDX test results. Predicting diagnoses prospectively poses significant challenges due to dataset limitations, imbalanced classes, and assumptions regarding the underlying pathophysiology. Nevertheless, there is an increasing amount of data suggesting that DNNs have the potential to enhance diagnostic accuracy (Maleki Varnosfaderani and Forouzanfar, 2024). Incorporating DNNs into diagnostic workflows could enhance decision-making, especially in scenarios requiring reassessment or complementary investigations.

## 2 Data and methods

The data used for this study has been previously presented in Nastasi et al. (2025).

### 2.1 Data

The dataset included 351 patients (3 patients were excluded for lack of data) who underwent at least one electrodiagnostic (EDX) assessment in the intensive care setting over a decade, from 2010 to 2019, at a tertiary pediatric referral center (Great Ormond Street Hospital, London). The patients’ ages spanned from 3 days to 17 years. They exhibited various neurological and clinical problems like respiratory distress, hypotonia, generalized weakness, or localized weakness. Respiratory support was necessary for 30% of the cases, and 11% of the patients were born prematurely. Among these patients, 33% passed away while in intensive care or shortly thereafter. They underwent one or more of the following test: nerve conduction studies (NCS), electromyography (EMG), repetitive nerve stimulation, and stimulated single-fiber EMG. Studies were conducted on multiple motor and sensory nerves, with an index value derived for compound muscle action potential (CMAP) amplitude, motor nerve conduction velocity, sensory nerve action potential (SNAP) amplitude, and sensory conduction velocity. This index was an averaged z-score within each subgroup. EMG was performed on one or more muscles, with results categorized by lumbar, cervical, and bulbar regions, which were assessed as normal, neurogenic, myogenic, or indeterminate. Spontaneous muscle activity was graded on a scale from 0 to 10, with 0 indicating no activity and 10 indicating florid activity. Routine nerve stimulation and single-fiber electromyography (SF-EMG) results were categorized as normal, abnormal, or indeterminate. A final diagnosis was established by examining clinical notes that detailed symptoms and test results, leading to patient classification into the following categories: non-primary neuromuscular disorder, neuropathy, neuronopathy, myopathy, neuromuscular junction disorder, and critical illness neuromyopathy.

### 2.2 Preprocessing of data

In preparation for data analysis, numerical features were standardized to achieve a mean of 0 and a standard deviation of 1, thus removing biases caused by varying feature scales. These numerical features included age and the indices for motor and sensory amplitude as well as conduction speed. Categorical features were converted into numerical form using one-hot encoding (Goodfellow, 2016). This method transforms categorical information into binary vectors, assigning each category to a vector with all elements as 0, except for a single 1 marking the particular category’s location. The categorical features included gender, respiratory support, mortality during or following intensive care, and electromyography records. The target data, representing diagnosis groups, displayed a skewed distribution (Non-primary neuromuscular: n=159; Neuropathy: n=45; Motor neuronopathy: n=55; Myopathy: n=49; Neuromuscular junction disorder: n=18; and Critical illness neuromyopathy: n=28). To mitigate class imbalance, class weights were computed based on the inverse frequency of each class, complemented by a weighted resampling technique. This approach balanced the dataset by increasing the samples of underrepresented classes and reducing those of overrepresented classes.

### 2.3 Visualization of data

The resampled dataset was used in a t-SNE (t-distributed stochastic neighbor embedding) analysis. This method is good at finding patterns in complex data, even when the data does not follow a normal distribution. It works by reducing the data from many dimensions down to just two (or three), making it easier to see and understand. The results were shown in two scatter plots. In the first plot, each point was colored based on its class label, i.e the disease subgroup. In the second plot, we used K-means clustering to group the points, and each cluster was colored according to the most common class label within it. This helped to show how well the clustering matched the known subgroups.

### 2.4 Deep neural network training and validation

To better understand and classify the data, we used a DNN. The model was trained and evaluated using stratified K-fold cross-validation, a method that ensures a fair and balanced assessment across all groups in the dataset. Our goal was to develop a reliable system capable of accurately identifying patterns and classifying the data into multiple categories.

In this study, we developed a neural network model for multi-class classification using stratified K-fold cross-validation to ensure robust evaluation. The dataset was randomly shuffled and divided into five equal-sized subsets (folds), with each fold preserving the overall class distribution of the original dataset. This stratification ensured that approximately 20% of the samples from each diagnostic category were included in every fold, allowing the model to be trained and validated on balanced and representative data splits. A fixed random seed was applied to ensure reproducibility of the results. For each fold, the training (80%) and validation (20%) sets were derived, and a feedforward neural network model was built using the TensorFlow Keras API. The model architecture included two hidden layers with 128 neurons each and a third hidden layer with 64 neurons, all utilizing ReLU activation. The output layer employed a softmax activation function to produce probabilities for each class. The model was compiled with the Adam optimizer and sparse categorical cross-entropy as the loss function, with accuracy as the evaluation metric. Each fold was trained for 20 epochs with a batch size of 32.

The predictions for each fold’s validation set were collected, and the overall model performance was assessed by aggregating predictions across folds. A classification report was generated to evaluate precision, recall, and F1-score for each class, while a normalized confusion matrix was visualized to analyze misclassifications. Additional metrics, including accuracy, multi-class ROC-AUC (using a one-vs-rest approach), and precision–recall AUC, were calculated to provide a comprehensive evaluation of the model’s performance.

**Fig. 1:**
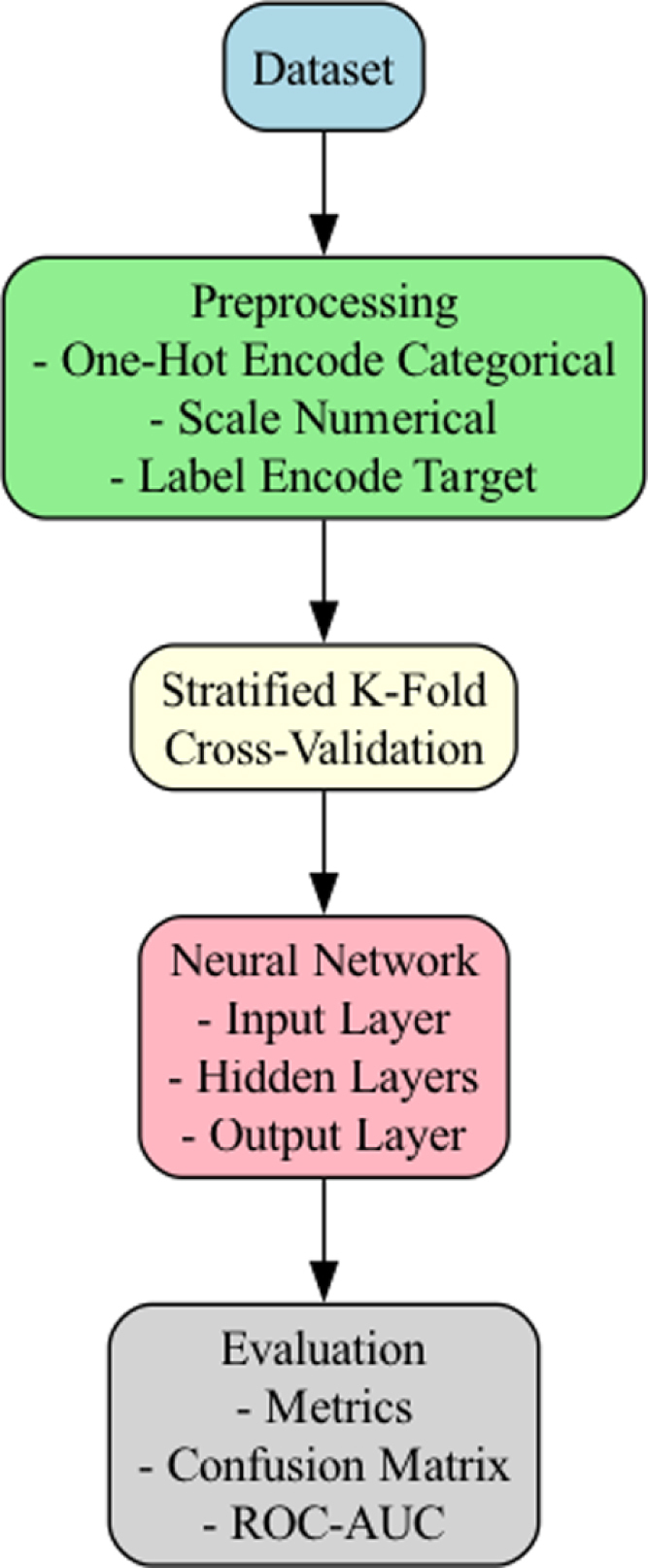
A pipeline of the data analysis process. Preprocessing consisted of encoding both the categorical and numerical variables as well as encoding the output variable (target variable). As the data was of modest size, splitting of the data (for validation) was done prior to training the DNNs. The averaged outputs from the validation (fold) was evaluated using different metrics.

**Fig. 2:**
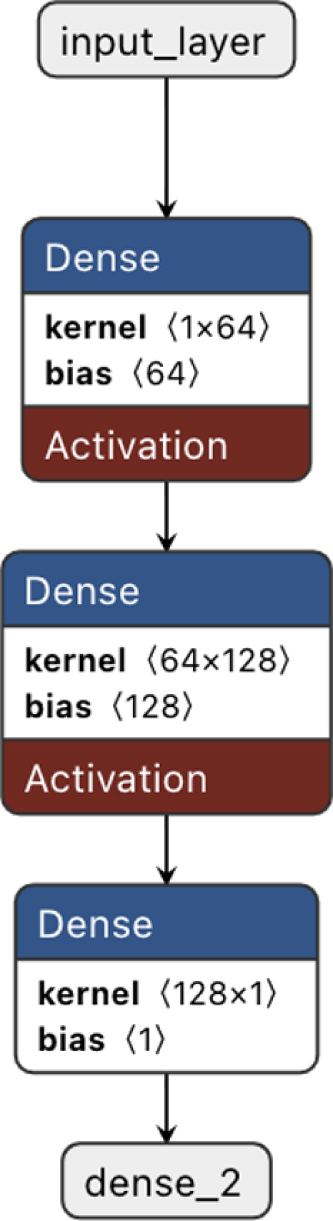
The DNN consisted of 3 layers with 64 and 128 intermediate neurons in each layer. The model was compiled with the Adam optimizer and sparse categorical cross-entropy as the loss function, with accuracy as the evaluation metric.

The approach ensured rigorous evaluation by cross-validation while maintaining consistency in the preparation and training of the neural network model, See Fig. 1, Fig. 2 for a schematic of the pipeline for the data analysis and also for the structure of the DNN.

### 2.5 Coding and statistical analysis

The analysis described in this study was conducted in Python 3.11.5, using the scikit-learn 1.6.1 and TensorFlow 2.16.1 libraries. Scikit-learn was utilized for data preprocessing, implementing stratified K-fold cross-validation, and calculating various evaluation metrics, while TensorFlow’s Keras API was employed to design, train, and validate the neural network models. The complete codebase for this study, including all preprocessing steps, model training, validation, and evaluation procedures, is publicly available at https://github.com/gercoo/DNN_EDX.

## 3 Results

The findings from the analysis detailed in the methods section will be shown in Sections 3.1, 3.2.

### 3.1 Visualization of data

**Fig. 3:**
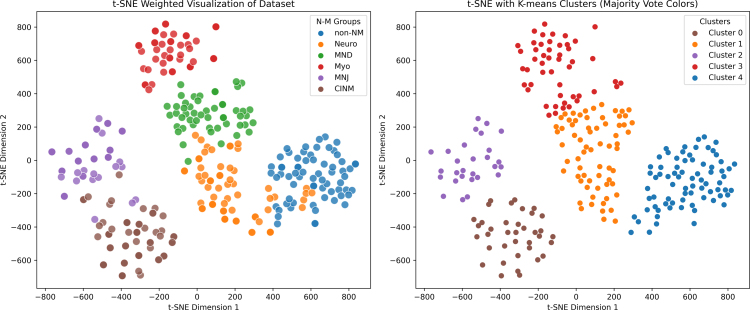
t-SNE was utilized to visualize clusters within the dataset. On the left, there is a 2-dimensional t-SNE plot, with each point colored according to its corresponding target group. The plot on the right depicts the identical t-SNE plot, where K-means clustering has been applied, revealing five clusters. Patients with neuropathies and MND were clustered together. Differentiating these groups using EDX data is challenging, mainly because the key distinctions lie in sensory nerve studies, which are frequently hard to conduct in an intensive care environment. Nonetheless, it is noteworthy that the majority of diagnosis groups were identified without any prior classification of the data. The findings indicate that a neural network can be effectively trained on this data with minimal risk of overfitting, as multiple groups were successfully distinguished using an unsupervised method.

We used t-SNE to investigate the structures of the data used in this study. After preprocessing the data (as described in the Section 2) t-SNE was performed, projecting the data into 2 dimensions such as to optimize the clustering of the data into subgroups. The number of clusters were identified using K-means clustering and Siloutte analysis (Rousseeuw, 1987), see Fig. 1. There were 6 diagnosis groups, as seen on the panel to the left, while clustering identified 5 of these groups as patients with MND and Neuropathies were placed in the same group. This analysis highlights the richness of the dataset, as t-SNE, used here as a blind categorizer, was able to uncover several disease groups based solely on differences in their EDX data profiles (see Fig. 3).

### 3.2 Deep neural network training and validation

**Fig. 4:**
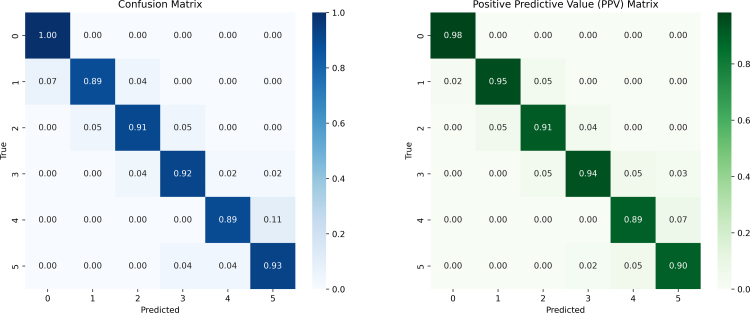
From the training and validation of the DNN a confusion (classification performance) and PPV matrix was created to display the performance and diagnostic accuracy of the DNN. It can be noted that all diagonal terms are > 85%. The diagnosis groups were numbered as: 0—Non-primary NM, 1—Neuropathy, 2—MND, 3—Myopathy, 4—NMJ, 5—CINM.

**Fig. 5:**
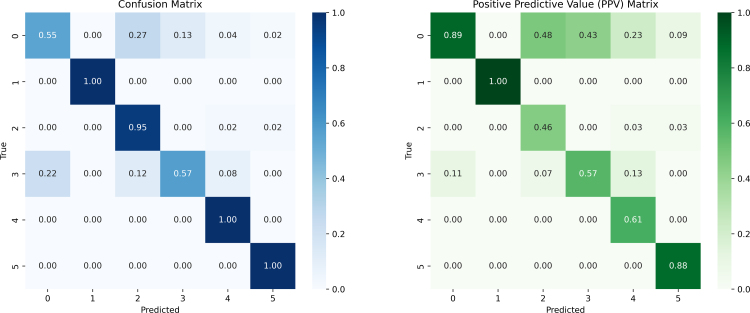
We used the clinical predictions of the diagnosis group to create a confusion (classification performance) and PPV matrix to display the performance and diagnostic accuracy. There is an uneven distribution of the diagonal entries evident for both matrices. The PPV matrix indicates several diagnostic groups that could not be predicted with a high level of certainty (NMD, Myopathy and NMJ), several of these cases were found out to have been misdiagnosed with the non-primary neuromuscular disease group. The diagnosis groups were numbered as: 0—Non-primary NM, 1—Neuropathy, 2—MND, 3—Myopathy, 4—NMJ, 5—CINM.

The pre-processed data was also used to train a DNN, as described in Section 2. The classification results were evaluated using both a confusion matrix (to assess overall classification performance) and a positive predictive value (PPV) matrix (to estimate diagnostic reliability). In both matrices, the highest probabilities were concentrated along the diagonal, indicating correct predictions, with values exceeding 84% for the confusion matrix and over 89% for the PPV matrix (see Fig. 4). The data were annotated by experts in pediatric neurophysiology (GC, LN, JD) according to standardized diagnostic criteria routinely employed in clinical settings, as referenced in Nastasi et al. (2025). This enabled a direct comparison between the diagnostic performance of expert clinical assessment and that of the DNN. While both approaches exhibited broadly concordant diagnostic patterns, the DNN produced a more evenly distributed set of predictions across the differential diagnostic categories, indicating a potentially wider diagnostic scope, see Fig. 5. The accuracy of the DNN (0.952), the ROC-AUC (0.999) and Precision-Recall AUC (0.990). The precision, recall and f1 score for each diagnosis group is shown in Table 1.

**Table 1:** The following metrics for estimating accuracy were determined during the training validation of the DNN. All values are above 0.89 indicating a good fit to the data. Note that the validation was done using cross-validation to reduce the risk of overfitting the training model.

Precision metrics for DNN				
	Precision	Recall	f1 score	N
Non-primary NM	0.9822	1	0.9910	166
Neuropathy	0.9524	0.8889	0.9195	45
MND	0.9091	0.9091	0.9091	44
Myopathy	0.9375	0.9184	0.9278	49
NMJ	0.8947	0.8947	0.8947	19
CINM	0.8966	0.9286	0.9113	28

## 4 Discussion

This study investigated the use of DNNs for predicting clinical diagnoses in pediatric intensive care patients with neuromuscular disorders. Specifically, we applied DNNs to classify patients into diagnostic categories and evaluated their performance and diagnostic accuracy. While previous studies have utilized various machine learning methods—such as Random Forests, Decision Trees, Logistic Regression, and Support Vector Machines (Maleki Varnosfaderani and Forouzanfar, 2024)—deep learning, particularly DNNs, has demonstrated significant promise. These methods are versatile, require less domain-specific expertise, and have broad applicability (Craik et al., 2019, LeCun et al., 2015).

The dataset, previously described in Nastasi et al. (2025), demonstrated clinically acceptable predictions of final diagnoses. The primary objective of this study was to assess whether a DNN could effectively leverage this information to achieve diagnostic classifications comparable to clinical standards. The clinical classification was not validated on a separate set of data. However clinical classification, rooted in decades of expertise correlating EDX data with neuromuscular disorders, served as the benchmark (Hafner et al., 2019, Pitt, 2011, David and Jones Jr., 1994). The study aimed to have the DNN extract and utilize this embedded knowledge using generalizable techniques.

Before training the DNN, the inherent structure of the data was evaluated using t-SNE (van der Maaten and Hinton, 2008) for clustering analysis. K-means clustering revealed five groups instead of the six diagnostic categories. Variability in cluster counts was noted due to the inherent randomness of the algorithms (e.g., gradient descent in t-SNE and centroid initialization in K-means) (David and Vassilvitskii, 2006). This variability was mitigated by running multiple iterations, averaging cluster counts, or employing the K-means++ algorithm to ensure dispersed centroid selection. NMJ and CINM clusters were adjacent, despite their distinct clinical features: NMJ is associated with pre-admission fatigue and severe weakness, whereas CINM is linked to post-intensive care presentations with prolonged stays and respiratory support—factors not captured in the dataset (Kasinathan et al., 2021, Marrero et al., 2020, Trojaborg, 2006, Sarikaya Uzan et al., 2022). These two clusters shared similar nerve conduction study patterns, such as normal sensory amplitudes and potentially low motor amplitudes, but other specific diagnostic tests were key to differentiating them. Similarly, neuropathy and MND were identified as one cluster, reflecting similar EMG patterns, apart from the absence of sensory abnormalities in MND—a difference that is challenging to evaluate in intensive care environments (Bouche et al., 1999). Despite these complexities, the analyses successfully demonstrated the dataset’s intrinsic clustering patterns.

Given the dataset’s modest size, careful partitioning into training and validation sets was necessary. Ensuring both sets included all diagnostic groups was particularly challenging due to class imbalance. This issue was addressed through cross-validation, wherein the DNN was trained on different data subsets, and performance was averaged across folds (Hastie, 2009). Cross-validation reduced the risk of overfitting and enhanced the robustness of the results. Training with and without cross-validation yielded comparable outcomes, although cross-validation provided greater confidence in mitigating overfitting.

The DNN architecture was optimized by varying the number of layers and neurons per layer. A configuration of three layers with 64 or 128 neurons per layer was used, as further adjustments did not significantly improve accuracy. This consistency reinforces the robustness of the findings, aligning with the clustering patterns observed in the t-SNE analysis (Goodfellow, 2016).

The DNN demonstrated high precision, especially when compared to the predictive power of EDX-based clinical diagnoses. While clinical diagnoses were less accurate for three diagnostic groups (MND, Myopathy and NMJ), the DNN achieved relatively high accuracy across all groups. This highlights the potential utility of DNNs in handling complex datasets in healthcare. It is important to note, however, that clinical diagnoses rely on broader contextual information and may remain robust across different patient populations, whereas the DNN’s performance might vary with changes in data distribution. Nevertheless, the study underscores the value of DNNs as diagnostic adjuncts, particularly in cases where discrepancies between the DNN and clinical diagnoses prompt further investigation or additional diagnostic testing.

EDX tests were selected for each patient according to clinical suspicion at the time of evaluation. This approach alters the pretest probability of various diagnoses, which may influence the diagnostic outputs of the DNN. As the study did not employ a standardized test battery across all patients it is not possible to assess the unbiased diagnostic yield or predictive accuracy of the EDX tests themselves. However, this reflects routine clinical practice, where test selection is typically hypothesis-driven and tailored to the suspected pathology.

This study applied DNN methods to predict diagnoses based on EDX-test results and compared the model’s performance to that of standard clinical assessment. A key challenge in applying DNNs in this context is the high risk of overfitting, which stems more from the architectural complexity and large parameter space of the model than from the characteristics of the dataset itself. To address this, we implemented several regularization techniques and design constraints, resulting in stable and generalizable performance on the held-out test set. While other machine learning (ML) approaches could also be explored for diagnostic classification and comparative evaluations would be valuable, the primary objective of this study was to assess the predictive accuracy of the DNN relative to expert clinical interpretation. Furthermore, unsupervised clustering using a “blind” t-distributed stochastic neighbor embedding (t-SNE) approach partitioned the data into five distinct clusters: four corresponding to different neuromuscular disease categories, and one comprising patients with either neuropathy or motor neuron disease. This suggests that the DNN’s classification behavior is not solely a result of overfitting. Nonetheless, external validation on larger, independent datasets is necessary to evaluate the broader applicability and generalizability of the model.

Despite being conducted on a small dataset, the model achieved high precision in predicting final clinical diagnoses. Future research should extend this approach to other patient populations to determine whether DNNs maintain performance levels comparable to clinical predictions. Incorporating DNNs into diagnostic workflows could enhance decision-making, especially in scenarios requiring reassessment or complementary investigations.

## CRediT authorship contribution statement

**G.K. Cooray:** Conceptualization, Design of the study, Data analysis, Statistical analysis, Preparing tables and figures, Writing – review & editing. **L. Nastasi:** Data analysis, Writing – review & editing. **D. Motan:** Data analysis, Writing – review & editing. **J. Deeb:** Conceptualization, Design of the study, Data analysis, Writing – review & editing.

## Ethical approval

The project was registered and approved by the Trust internal review board and audit committee at Great Ormond Street Hospital, London (registration number 3344).

## Declaration of generative AI in scientific writing

Statement: During the preparation of this work the author(s) used ChatGPT 4 to nuance the language. After using this tool, the authors reviewed and edited the content as needed and take full responsibility for the content of the publication.

## Declaration of funding

No specific funding was received during the study.

## Declaration of competing interest

None.
